# A definition-by-example approach and visual language for activity patterns in engineering disciplines

**DOI:** 10.1371/journal.pone.0226877

**Published:** 2020-01-10

**Authors:** Mario Janke, Tobias Kuschke, Patrick Mäder

**Affiliations:** Software Engineering for Safety-critical Systems Group, Technische Universität Ilmenau, Ilmenau, Germany; Wright State University, UNITED STATES

## Abstract

Modeling tools are well established in software development. A model is the result of a series of modeling activities. The ability to recognize when a user is working on a certain modeling activity opens up a range of possibilities for context-sensitive support. One possible way to support the user is offering the auto-completion of the current task. The recognition of modeling activities is typically carried out by matching event patterns against events emitted by a user’s editing operations. A user that intends to add or customize auto-completions must be able to easily understand and create activity definitions. However, defining the currently required complex event patterns is a challenging and error-prone task even for a person with an intensive knowledge of event-processing languages. In this paper, we propose the visual definition language VisPaRec accompanied by a method that allows creating activity definitions in a semi-automated and graphical way. We evaluate our visual definition language in a comparative user study against the generic event-processing language Rapide. We found that the proposed visual representation increases comprehensibility while reducing time for constructing and modifying activity definitions significantly.

## 1 Introduction

Automating repetitive tasks in software engineering processes has become essential for developers. Apart from increasing the efficiency of the development process, it can also improve the quality of the developed product by obviating human errors. For example, software developers, experts and novices alike, benefit from simple automations like auto-completion in an IDE [1]. Researchers demonstrated that automation can be beneficial for graphical modeling tasks as well [2–5]. There are plenty of opportunities to support a developer in recurring activities during model-driven architecture and design (e.g., model refactorings). However, the recognition of these activities is not a trivial task since they include a number of different, related editing operations. Complex event processing (CEP) [6] allows for the recognition of such activities and is the technology we apply for this and previous work. In previous work, we used complex patterns of editing events to update traceability information automatically upon recognizing users’ modeling activities [7] and successfully brought a visual language approach to traceability analysis [8–10]. In a succeeding project, we studied how software modeling activities can be recommended while a developer is editing UML models [11] and how such recommended activities can then be auto-completed [12–14]. Both proposed approaches build upon activity definitions captured as event-processing rules. Reactive to every editing operation carried out by the user, an implementation of the latter approach recommends a ranked set of context-relevant modeling activities for auto-completion. The typical workflow of integrating activity pattern-based completion into a tool includes using a general-purpose CEP language or declarative programming language. Alternatively, simple patters can be hardcoded without a proper CEP framework. However, this comes with serious drawbacks in terms of maintainability and expandability. We have previous developed a prototype architecture of an auto-completion tool based on a textual CEP language, but we hypothesize that a specialized visual language can increase the applicability of this and many other approaches. In general, the usefulness of such automation highly depends on their customizability to users’ needs. However, defining the required event-processing rules is a challenging task because very specific knowledge of the processing platform and its declaration language is required.

This paper presents the visual language VisPaRec accompanied by a definition method allowing non-CEP-experts to create and maintain activity definitions in a semi-automated way. We see multiple application scenarios where lowering the effort and knowledge necessary to work with activity definitions can benefit developers in the software industry. On one hand, tool vendors of model and code editors can develop CEP pattern based support more easily. On the other hand, modelers using modeling tools directly benefit by being able to customize and define their own activity definitions. The modelers may or may not be active programmers, but have full insight into their personal working patterns and customization wishes. Our approach aims to aid users in defining their own patterns and by that supporting their individual workflows. In this sense, pattern specification should require as little knowledge about programming and CEP as possible. Patterns should be comprehensible at a glance, easily maintainable and expandable. Through the definition-by-demonstration method proposed in this work, users can create activity definitions by demonstrating the desired activity within their modeling environment rather than being requested to write complex event processing rules. Thereby, technical and tool-specific aspects are abstracted and mostly hidden from the user.

The remainder of this paper is structured as follows. Section 2 reviews related work on complex event patterns, defining model transformations and programming by example. Section 3 describes the concepts of modeling activity definitions and their relation. Section 4 introduces VisPaRec, the visual definition language that we developed for specifying modeling activity definitions, followed by an analysis and comparison of selected event processing languages. Section 5 presents how VisPaRec can be embedded into a “by-demonstration” approach that automates large parts of creating a definition. Section 6 describes a user experiment that we conducted to evaluate comprehensibility, applicability and usability of VisPaRec. Finally, Section 7 concludes our work and outlines future research.

## 2 Related work

Supporting development activities in modeling-based environments is a relatively novel research topic. To the best of our knowledge, there exist no approaches that support users in defining their own activities and in employing them in modeling environments for auto-completion. There do, however, exist generic solutions for supporting users in the definition of complex event patterns and model transformation rules. Complex event processing, in general, is a technique to infer high-level knowledge from streams of low-level events. A core principle is the agglomeration of the simple input events into more structured agglomerations called complex events. Another characterizing feature are queries against these events, simple and complex alike. Usually, incremental evaluation is a key feature. Typical applications range from monitoring tasks, such as evaluating sensory data and network monitoring, over gesture detection to business rule engines.

### Textually defining complex event patterns

Eckert et al.’s [15] comprehensive survey shows that, driven by varying user needs as well as strengths and weaknesses of the underlying engines, a rather high number of textual CEP languages have been proposed in the past. We exemplarily discuss three languages that follow different approaches in their design. Given the large variation in language features in the early days of rule processing, *Rapide* [16, 17] was proposed as a language independent of an underlying engine. It has a strong mathematical foundation and was designed as a general language framework. Rapide consists of a comprehensive and expressive set of rule features that has inspired CEP engines since and that is supported by most CEP engines today. The *Drools Rules Language* (DRL) [18, 19] is one of many production rule languages. It is tightly coupled with its host language Java. In DRL, persistent state is represented as Java objects called *facts*, which are stored in a *Working Memory*. Pattern matching happens through rules that specify facts and conditions that single or multiple facts must fulfill. *Esper EPL* [20] is a data stream query language. Its language design originates from the database query language SQL. Esper EPL abstracts the input as a data stream of tuples. These streams can be queried much like a relational database. Timing as well as sequencing constraints can be formulated in a high-level syntax. Additionally, Esper EPL allows combining data stream queries with pattern matching. We compare the part of these languages’ syntax that is relevant to the presented approach in Section 4.3.2.

### Visually defining complex event patterns

In the field of complex event processing, a number of graphical editors, both proprietary tools and academic approaches, for defining patterns and rules exist. The *SocEDA* project [21] offers an editor for translating high-level definitions of complex event patterns into Esper EPL. The tool supports the pattern definition visually but also requires the user to write code of the underlying event-processing language. The visual representation of a pattern in SocEDA does not contain all information, but rather represents some of it merely within the code. Nowak et al. [22] present an editor based on the *Palantir Government* platform for creating rules in the event-processing language *JESS*. Similar to SocEDA, the visual representation does not include all the relevant information. The editor only presents additional information in a panel when requested by the user. Both approaches reduce the visual load by omitting some information and only showing it on demand. While this is effective for a modeling tool, it does not enable visual comprehension of patterns at all. *Strelka* is a graphical editor for defining rules developed by the *REWERSE Working Group I1* [23]. The tool uses the UML based rule modeling language *URML* that allows users to visually model different types of rules based on UML class models. Boubeta-Puig et al. [24] describe a user-friendly, model-driven approach for specifying complex event patterns. Similar to our proposed method, users are able to design graphical models specifying event patterns. These models are then transformed into platform-specific event-processing code by the use of a corresponding model-to-text transformation. Their editor *MEdit4CEP* provides all instruments that are necessary to express conditions for recognizing modeling activities. However, even low-level language elements like logical operators are displayed by separate visual elements, which leads to high visual complexity and clutter. In contrast to the first two approaches, MEdit4CEP and Strelka do encode rules completely visually. The drawback of this approach is that the visual pattern definitions become very complex even for small rules, since a lot of information that could be represented very compactly, is displayed in a very explicit way. We further investigate this issue in Section 4.3. The introduced editors allow defining event patterns and rules without explicit knowledge of a platform-specific event-processing language, but they all are developed from the viewpoint of complex event processing. Additionally, these approaches do not provide mechanisms for defining auto-completions of modeling activities.

### Defining model transformations

Sun et al. [25] presented an approach that allows users to demonstrate model transformations and to automatically derive transformation rules from this demonstration. In another study, Sun et al. [26] describe how layout information for model transformations can be captured during user demonstrations and effectively used later on. Biermann et al. [27] propose a framework for *in-place* transformations of EMF-Models. Based on their approach, Arendt et al. [28] developed the transformation language *Henshin*. In their work, they describe concepts like pre- and post-conditions of model states that have similarities to our work. However, transformation rules are intended to change models upon reaching defined states, whereas our approach regards edits, not state. Specifying the evolution of model elements comprising historic element states and restricting the timing of model changes are not the purpose of model transformation rules.

Graph transformation languages like Story Diagrams [29] can specify patterns similarly to transformation languages. A pattern matches a graph and applies the transformation to it, the outcome is a modified graph. This approach, therefore, cannot be used directly to specify an activity pattern. However, one could try to reinterpret the existing visual notations for graph transformations or augment them. Graph transformations only specify a state before and after the transformation. For the specification of activity patterns, continuous and possibly concurrent changes need to be specified.

### Programming by example

Raza et. al. [30] propose a solution to the problem of ambiguity in example-based programming. They use the least general generalization to describe a set of examples. The inherent ambiguity involved in such tasks is a highly important issue our approach faces as well. Their approach aims to express rather small changes using a multitude of examples and was evaluated using a Microsoft Powerpoint add-in.

### Conclusions

We conclude that there are approaches that support the graphical specification of rules. However, none of them provides a desirable solution to the problem of specifying event-processing rules in the context of modeling activity recognition. This is what we aim to accomplish with VisPaRec. VisPaRec is not intended to be a general-purpose EPL, but rather a domain-specific language for the representation of modeling activities that is easy to read and write for non-CEP-experts.

## 3 Concepts of modeling activity definitions

We aim to support a user in developing a model that comprises a set of *model elements* *E*. Typical model elements are packages, components, classes, interfaces, attributes, methods, associations, dependencies, and generalizations. The user is evolving this model through *editing operations* of the types add, modify, or delete. A modify operation changes attributes of an existing model element, e.g., the element’s name or type. This choice of operation types is extendable in nature and may be dependent on the application at hand. For specific applications, it might be useful to formulate more types. However, specific operation types like copying a model element or the act of moving one model element into another can be represented as a pattern of the fundamental types add, delete and modify. We denote the set of all editing operations performed by a user on a model as *O*. A *modeling activity* is then defined as a recurring set of editing operations carried out by the user on model elements.

**Fig 1:**
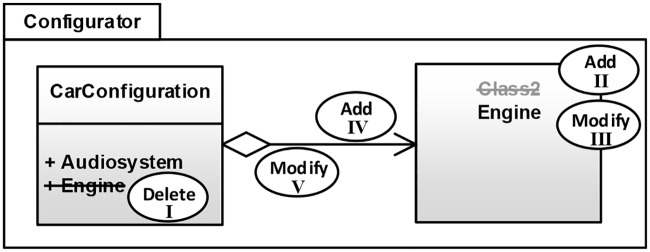
Editing operations for executing the activity “Extracting attribute as class”.

As a running example, we introduce a modeling activity that represents the well-known refactoring “Extract Class” (see Fowler [31]) on a UML class diagram. During this refactoring, an attribute of an existing class is extracted into an independent class. Fig 1 shows the modeling activity performed on an example model of a software system. As a precondition, a class must exist and contain an attribute. In the example, the attribute *Engine* of the class *CarConfiguration* is extracted. The ellipses represent editing operations belonging to the modeling activity. The existing attribute is deleted (I) and a new class is added (II) within the same package *CarConfigurator*. The new class will be renamed (III) according to the deleted attribute *Engine*. The activity is completed by adding a new association connecting the two classes (IV) and modifying it into a directed aggregation (V). The user may freely decide about the sequence of editing operations. Naturally, some limitations are imposed by the modeling environment, e.g., classes need to be added before they can be connected.

The event-based recognition of a modeling activity requires an *activity definition* in the set of all activity definitions T. An activity definition specifies: (1) a set of expected editing operations that have to be performed to complete the modeling activity, (2) a set of expected *element versions* V capturing expected states of model elements during the modeling activity, (3) constraints on model element properties captured in element versions $P_{V}$ and across element versions $P_{R}$, (4) constraints across editing operations and element versions $S_{R}$ restricting the operation sequence, and (5) an action to be executed upon recognition of the specified modeling activity. It is sufficient to store *values* of element properties in $P_{V}$, while $P_{R}$ needs *references* to multiple element versions. The action part of the definition depends on the usage scenario. There are numerous ways to react to a detected activity, but in the scope of this paper, we illustrate our approach within an auto-completion technique that aims to complete the modeling activity the user started. Information specified in the action part of an activity definition therefore comprises *completion actions* A to be executed automatically and *property defaults* D to specify values of editing operations to be auto-completed.

**Fig 2:**
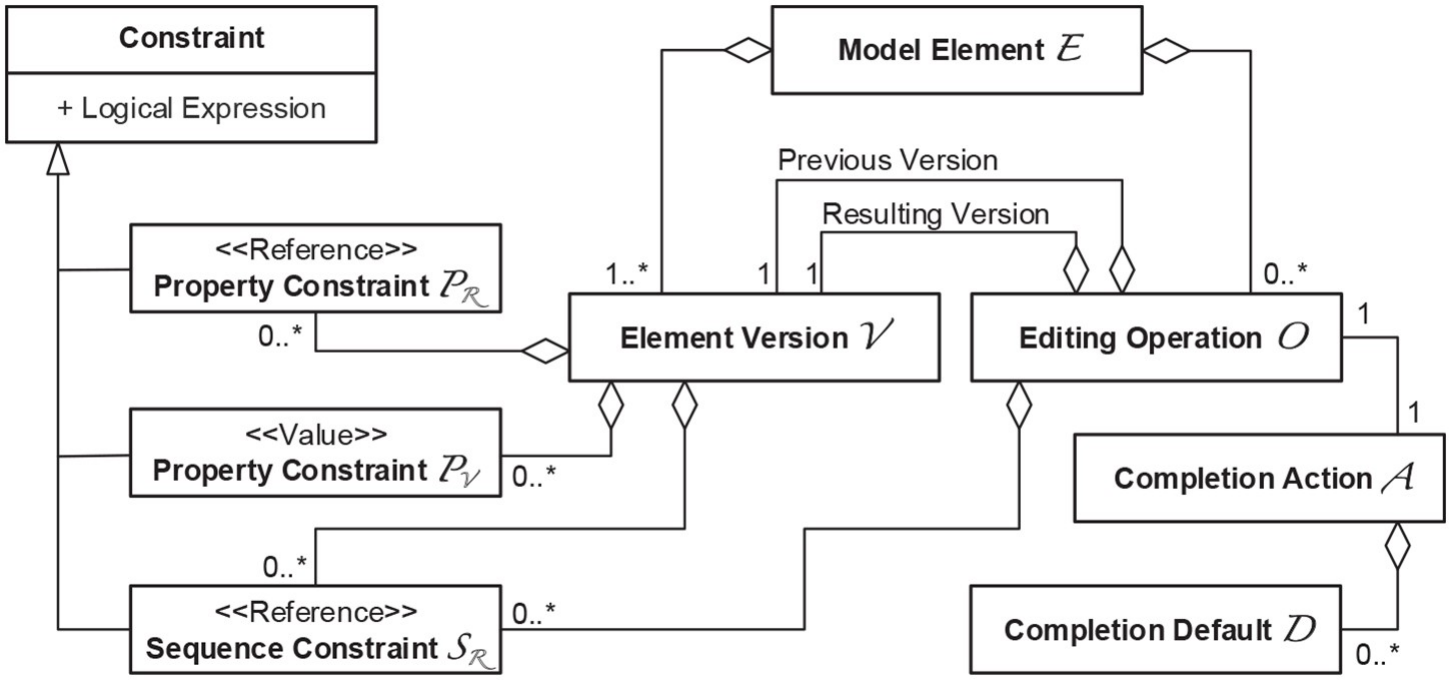
The meta-model for activity definitions.

Based on the introduced initial concepts, we developed a meta-model for activity definitions (see Fig 2). Whenever a rule matches, the result must be kept in a structured way to be usable for a possible auto-completion. It must specify editing operations already done, and how to complete the ones not done yet. An example is contained in the experimental replication package (accessible at https://dataverse.harvard.edu/dataset.xhtml?persistentId=doi%3A10.7910%2FDVN%2FFTPF3Z, cp. R1T on page 32) showing how to specify the necessary information using three arrays. Explicit specification is necessary in any general-purpose language.

## 4 VisPaRec—A language for activity definitions

Based on the meta-model introduced in the previous section, we developed a visual representation for each concept. We followed Moody’s principles for creating efficient visual software engineering notations [32]. Moody argues that the basis for a “good visual notation” is having a clear design goal that specifies what kind of information needs to be presented, what audience is using this information (theory of cognitive fit), and what visual dialect is suitable for that purpose. Moody lists fundamental principles that a notation should fulfill. Referring to the principles of semiotic clarity, perceptual discriminability, semantic transparency, visual expressiveness and dual coding, the basic notation elements should exhibit the following characteristics. They should correspond 1:1 to their semantic construct. Therefore, we mapped each meta-model element to an individual visual notation element while keeping their number cognitively manageable (graphic economy). Furthermore, notation elements should be clearly distinguishable from each other by using the full range and capacity of visual variables like shape, color and size. Also, text should be used redundantly to support graphic elements. In the following, we introduce the notation elements of our visual definition language and explain them on examples.

### 4.1 Language concepts

#### Model element (C1)

**Fig 3:**
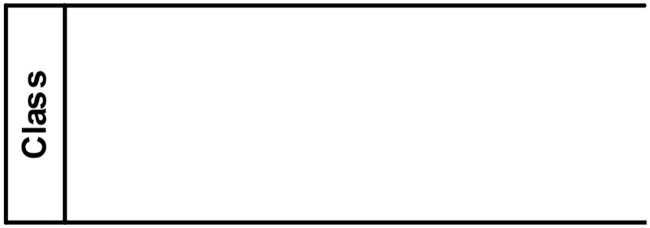
Visual representation: Model element (C1).

Each model element involved in an activity is represented by an open ended swim lane, much like in UML (see Fig 3). A vertical label on the left hand side of this swim lane contains the element type. The swim lane is a container that groups different versions of an evolving model element and the editing operations that transition from one version into the following.

*Decision*: Group versions of an element via swim lanes. *Rationale*: We see an object over its lifetime as a semantic construct on its own. Swim lanes help to reach the goal of representing each semantic construct 1:1 as they are a single notation element for a single construct. The commonly used alternative is to encode the edited model element through a guard of the object’s ID, which corresponds n:1 to the semantic construct. The swim lane concept also contributes towards graphic economy.

#### Element version (C2)

**Fig 4:**
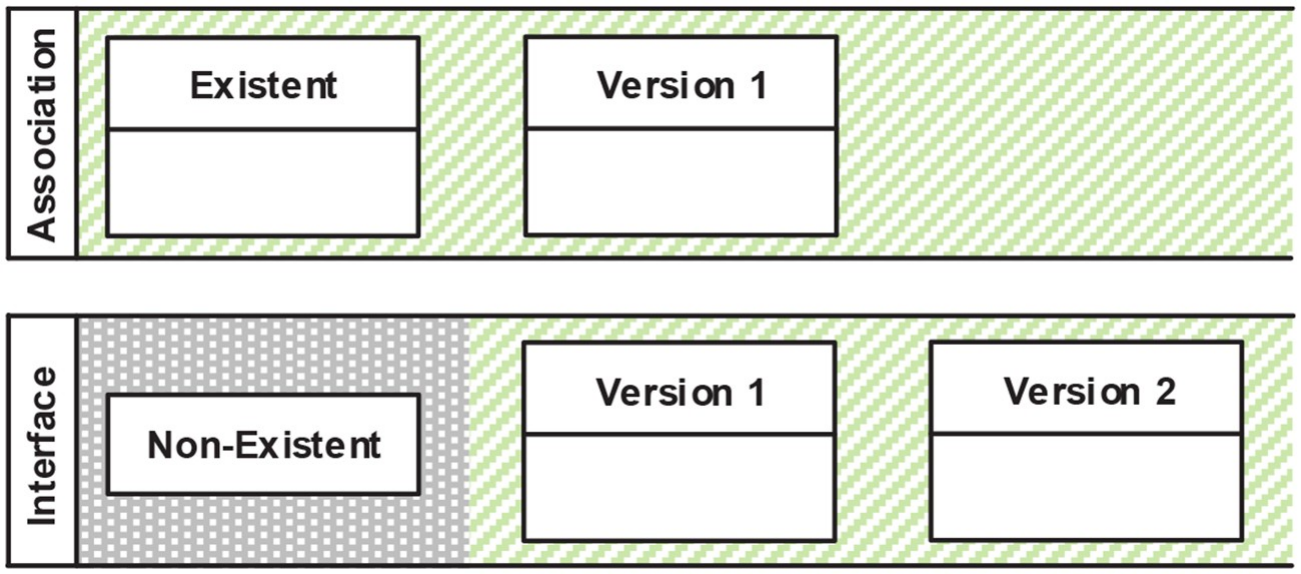
Visual representation: Element versions (C2).

An element version uses the notation of classes from a UML class diagram. We distinguish three types of element versions (see Fig 4): First, *Existent* specifies that a certain model element must exist before the modeling activity is started. Second, *Non-Existent* specifies that a certain model element may not exist before the start of an activity or after it is being deleted during an activity. Third, ordinary element versions specify certain states in the model element’s evolution. Different background colors are used within swim lanes to visually highlight the existence and the non-existence of a model element. Elements within the same swim lane have an implicit sequence constraint (see Sequence Constraints (C5)) among them, while no sequencing between swim lanes is enforced. This allows for more flexible patterns, as concurrency can be formulated.

#### Editing operation (C3)

**Fig 5:**
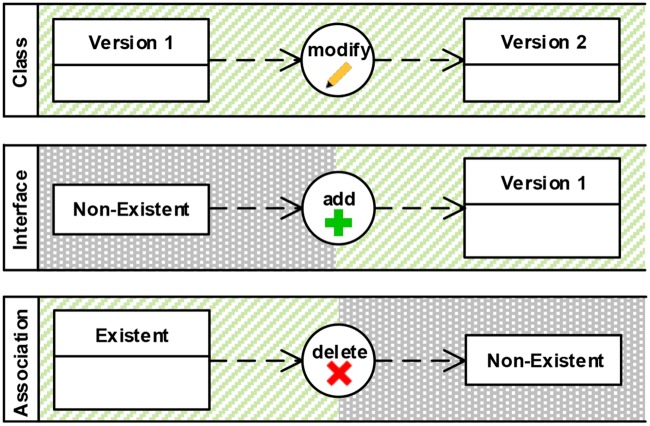
Visual representation: Editing operations (C3).

Editing operations are represented with a circular outline. According to the principle of dual coding, we visualize the operation types add, modify, delete, within the circle textually and additionally as a colored symbol (see Fig 5). An editing operation must be connected to a previous element version that specifies the state of the element before the operation is performed and to a resulting element version that specifies the element’s state after the operation. Two dashed arrows visualize these relations. The direction of those arrows points from the previous element version to the operation and from the operation to the resulting element version. The visual representation of model elements, element versions and editing operations all differ in shape and size. The graphical grouping of versions and operations into swim lanes is intended to clearly visualize the involved model elements and their evolution throughout a modeling activity.

*Decision*: Each operation type has its own icon distinguished by color and shape. *Rationale*: As the operation type has a huge impact on the interpretation of an edit, it must represent the types in a clearly distinguishable way ensuring semiotic clarity. Varying color and shape ensure perceptual discriminability. We expect this design to ease comprehension compared to all current solutions, which represent these concepts solely by a different name or id of the change type.

#### Property constraint (C4)

**Fig 6:**
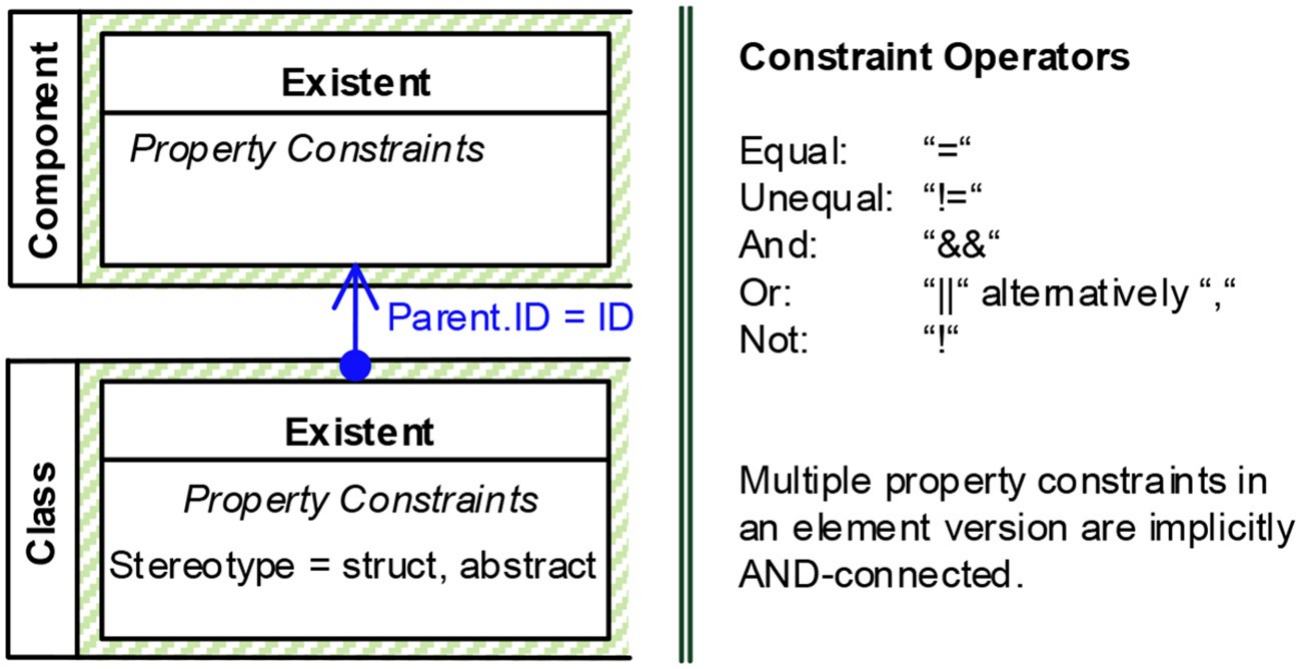
Visual representations: Property constraints (C4) (left) and possible constraint operators (right).

Property constraints specify values of element properties and are expressed as logical terms. To keep the graphical complexity manageable, we chose to not represent logical expressions in a graphical way. Instead, we specify these expressions textually by using operators like those shown in Fig 6. Property constraints are partitioned into static and dynamic ones. Static constraints *P*_*V*_ specify constraints on concrete values of element properties and are therefore defined within an element version. A very common static property constraint is the specification of the type of an element. Dynamic constraints *P*_*R*_ are defined as references across properties of versions of the same or different model elements involved in an activity. These references are visualized as directed connectors that point from the constrained to the referenced element version. Two elements having the same name, or one element being the child of another one are typical dynamic property constraints. The left hand part of Fig 6 shows the two visual representations of property constraints. Property constraints can be applied to restrict all element versions except those of the type *Non-Existent*. We do not check for syntactical or semantical mistakes within property constraints at modeling time. However, at a later point, the transformation into executable event processing rules (cp. Section 5.4) does check syntax.

*Decision*: Represent static and dynamic property constraints differently. *Rationale*: A fixed parameter and a reference between multiple model elements are semantically rather different. Separate visual representations help to uncover and visualize dependencies between model elements to the user and contribute to better perceptual discriminability of concepts.

#### Sequence constraints (C5)

Sequence constraints express timing and order conditions. They start and end at element versions or editing operations (see Section 3). We decided to visually distinguish sequence constraints from property constraints.

**Fig 7:**
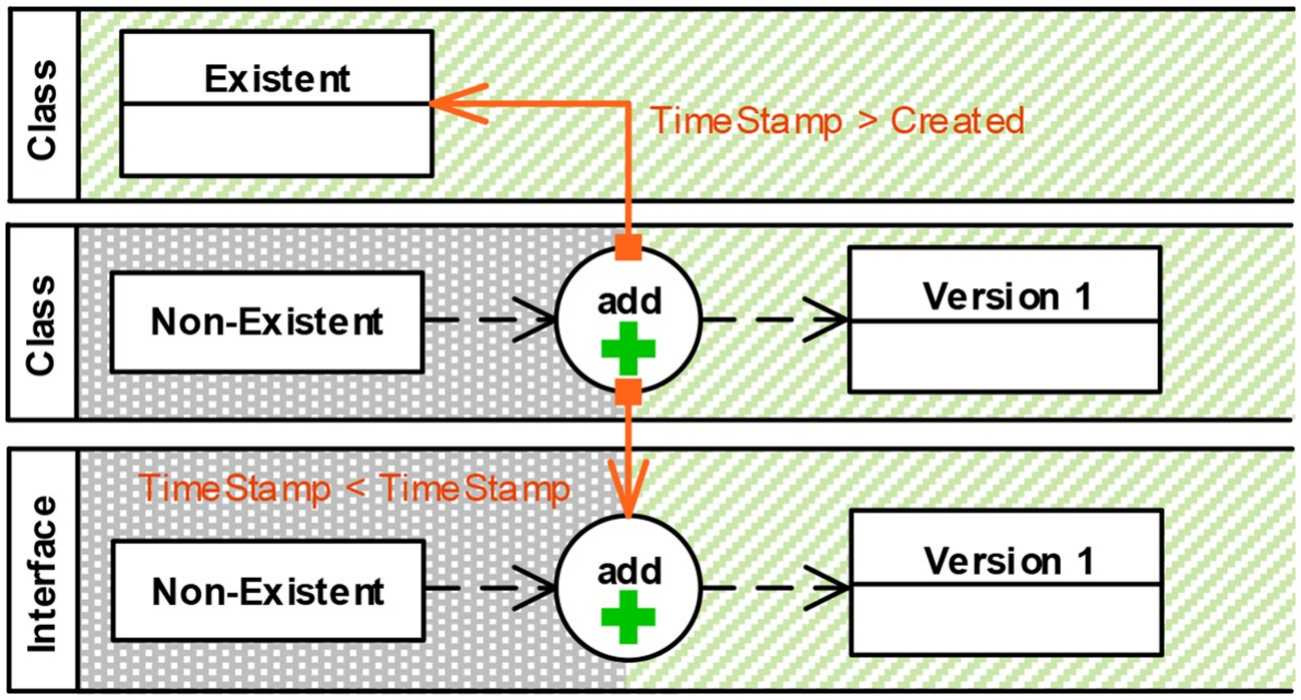
Visual representation: Sequence constraints (C5).

Sequence constraints use orange connectors instead of blue ones and squares as source symbols instead of dots. Fig 7 shows two examples of sequence constraints. The operator “>” means “*after*” and “*TimeStamp > Created*” (upper example) expresses that the editing operation must be performed after the model element has been created. In contrast “<” means “*before*” and the lower example specifies that the class has to be added before the interface is added.

*Decision*: Distinguish property constraints from sequence constraints. *Rationale*: We regard sequence constraints, which manage event order, and property constraints, which manage event content relations, as semantically different. Accordingly, we represent them differently increasing perceptual discriminability.

#### Completion actions (C6)

Completion actions serve as replacements for those editing operations that have not been performed manually. An activity definition comprises completion actions for all specified editing operations. Each completion action contains property value assignments that correspond to the property constraints of its editing operation. As such, it can be automatically retrieved from the editing operation definition. For that reason, completion actions are not explicitly expressed in a visual activity definition. Instead, each editing operation and the resulting element version visualize the corresponding completion action.

#### Completion defaults (C7)

**Fig 8:**
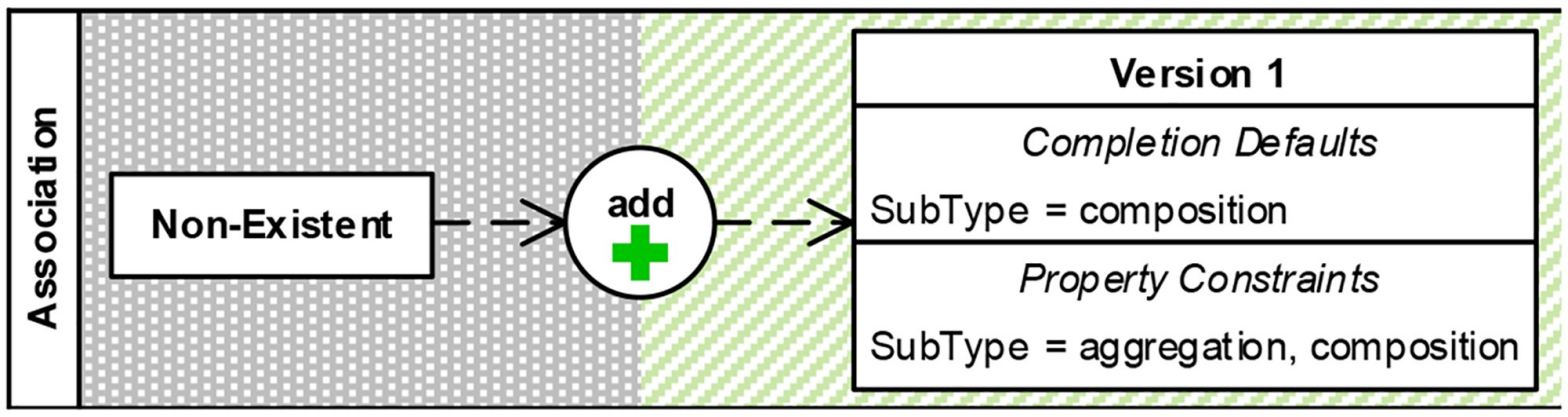
Visual representation: Completion defaults (C7).

Completion defaults specify default values for element properties that are applied when the auto-completion of a modeling activity is executed. Completion actions can comprise completion defaults that must be defined explicitly. We integrate the completion defaults into the resulting element versions of editing operations as shown in Fig 8. The example in the figure specifies that a user must add an association of subtype aggregation or composition to match. If this operation is auto-completed rather than manually executed, an association of subtype composition will be added.

### 4.2 VisPaRec’s expressiveness and scope

At the core, all EPLs are used to specify patterns by describing participating objects as *variables* and how they are related as *guards*, as well as what should happen whenever a pattern is matched as an *action* [33]. VisPaRec allows users to specify the variables in multiple different ways, tailored to the domain. Both, element versions as well as editing operations, serve as variables. As element versions are allowed to have arbitrary properties, they have full expressiveness. Sequence constraints, property constraints and model elements represent the guard concept. Model elements and sequence constraints, however, can only express specialized relations and are not as expressive as general guards. Property constraints can specify arbitrary relations between elements, and therefore are as expressive as the guard concept. This has the consequence that model elements and sequence constraints can be expressed as property constraints, but doing so results in massively harder to read and write descriptions. One can therefore see them as an abbreviating syntax construct. The completion actions are the equivalent of the action of a CEP pattern. One should note that the action of a CEP rule is only activated when the whole pattern is matched, while completion actions are applied whenever a single variable is matched. The behavior of completion actions is at least as expressive, as one can perform the desired action in the completion action of the object matched last. Table 1 summarizes the mapping between the basic CEP concepts and the visual concepts in VisPaRec implementing them. The table shows that for every CEP concept a VisPaRec element with the same expressiveness exist, concepts with full expressiveness are marked in italic. Therefore, VisPaRec can be used to express arbitrary complex events patterns.

**Table 1:** Mapping between CEP and VisPaRec concepts.

Basic CEP concept	Matching VisPaRec concept
variable	*element version*, editing operation
guard	*property constraint*, sequence constraint, model element
action	*completion action*, completion default

### 4.3 Comparison of event processing languages

**Fig 9:**
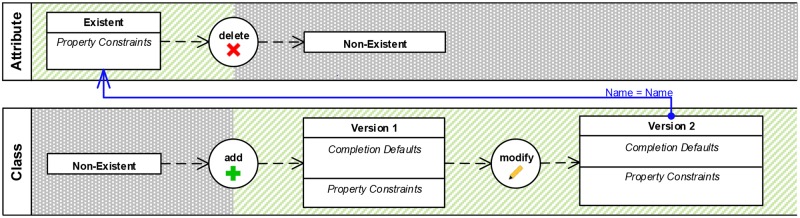
Example activity modeled in our VisPaRec language.

**Fig 10:**
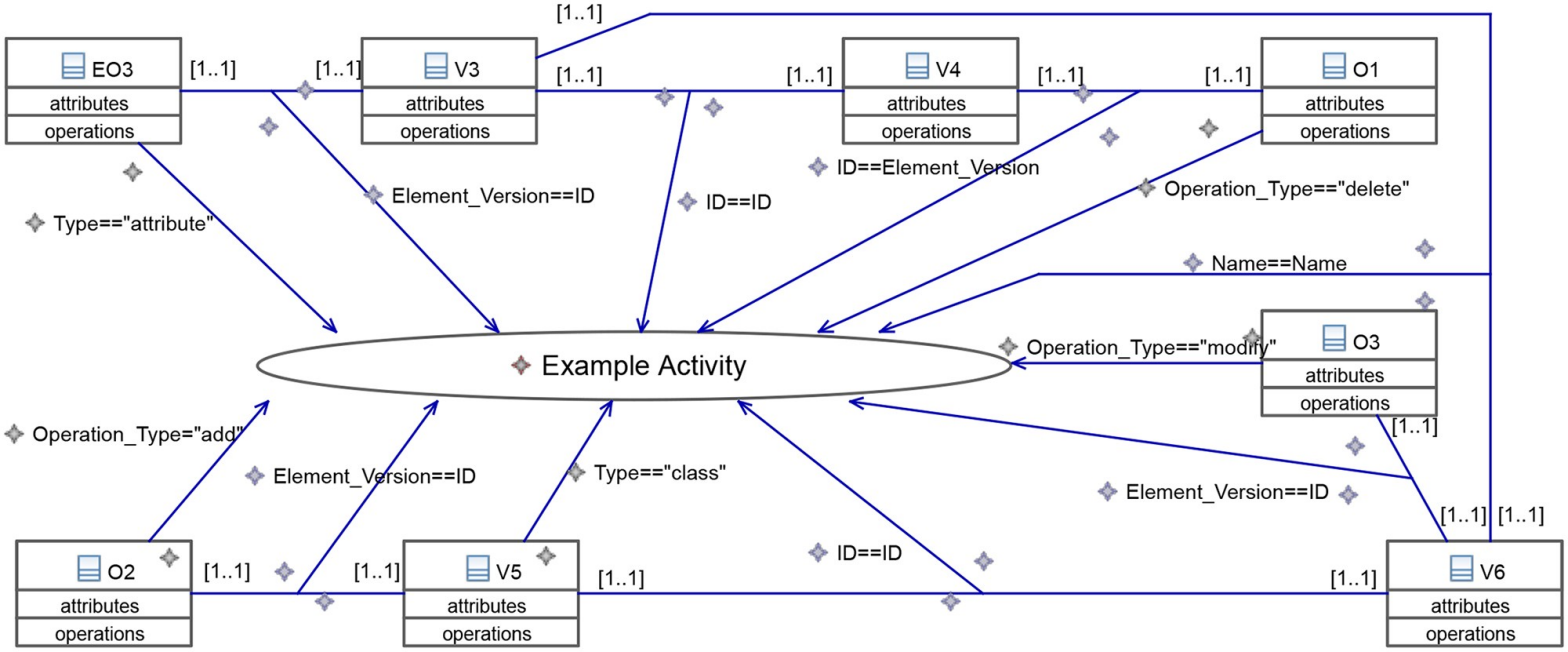
Example activity modeled in Strelka [23].

**Fig 11:**
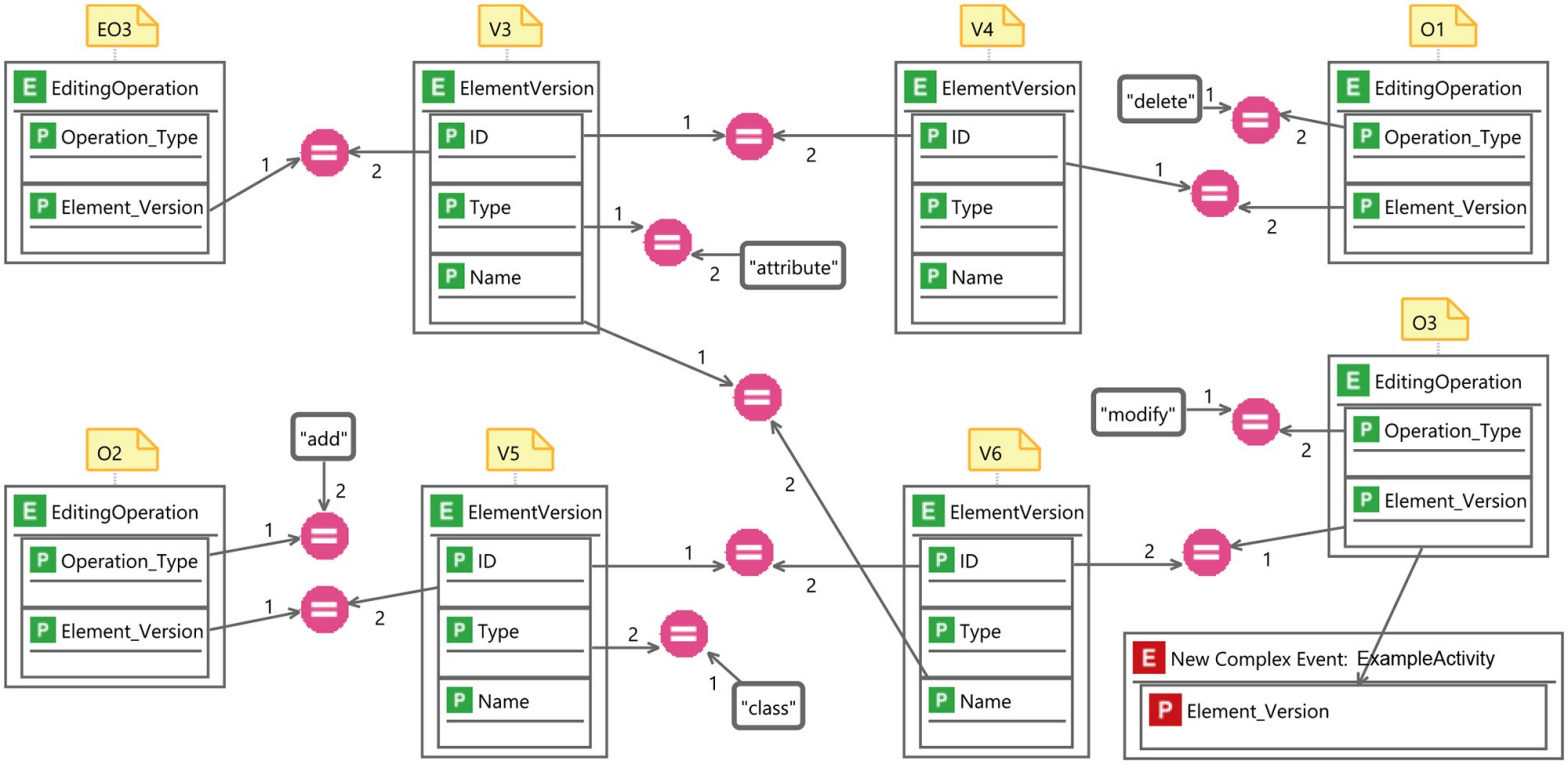
Example activity modeled in MEdit4CEP [24].

**Fig 12:**
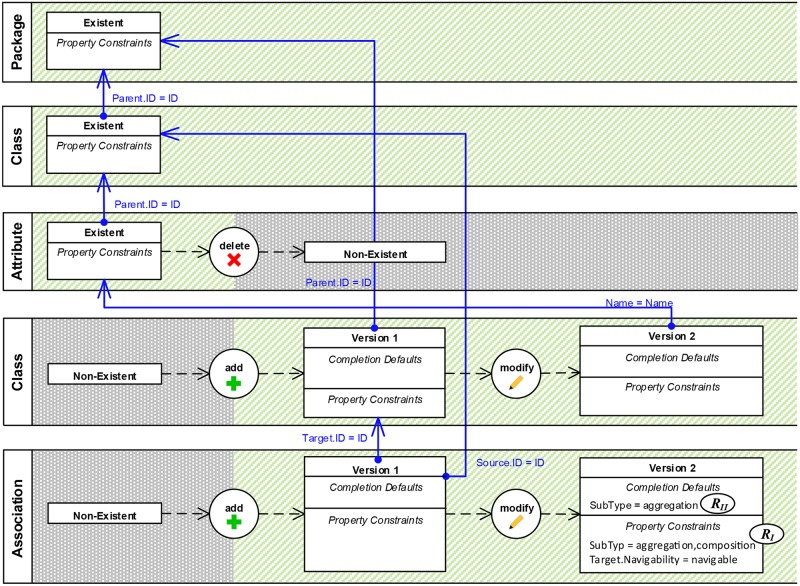
VisPaRec definition of the modeling activity “Extracting attribute as class”.

Now, we take a closer look into how different EPLs specify the basic CEP concepts discussed in Section 4.2 as well as the domain specific concepts described in Section 3. Fig 9 shows an example pattern in VisPaRec that we will refer to as the *example activity*. It is a simplified version of the pattern shown in Fig 12 that we reduced for space reasons. We formulate this pattern in various CEP languages in order to compare them with each other. The pattern matches when an attribute is deleted and a class is added, which is then renamed to the name of the deleted attribute. No additional explicit order between these events is enforced. Figs 9, 10 and 11 show the implementation of this activity in the respective languages.

#### 4.3.1 Evaluation of visual event processing languages

*Strelka*. Fig 10 shows how the example activity can be modeled with Strelka, using URML. The variables are represented by classes following the UML notation. The blue arrows pointing from a class to the rule are so-called *ClassificationCondition*s, specifying what a single class has to fulfill in order to fit the pattern. The blue arrows pointing from an association to the rule are *AssociationCondition*s, specifying how classes must be related in order to fit the pattern. These two concepts represent guards.

*MEdit4CEP*. The model depicted in Fig 11 represents the example activity modeled in MEdit4CEP. *Simple events* serve as variables and are represented as rectangles with a green “E” on the top left corner. If an event has properties, these are listed within the event’s rectangle as separate rectangles with a green “P” on the top left corner. Constant values are visualized as black rectangles with rounded corners and a broad border. Any comparison operator is represented by a single model element that is a pink circle containing the comparison symbol. It must have two incoming arrows to specify the arguments, which need to be ordered explicitly by the numbers “1” and “2”. Comparison operators together with constants represent the guard concept. Any pattern must contain a *complex event* that must have at least one property that is connected to a property of a simple event. Complex events are represented much like simple events, but use red labeling.

*Comparison*. The example shows that in Strelka and MEdit4CEP manyfold relations must be explicitly specified. This is the result of the very general nature of both languages. These relations could actually be formulated more intuitively and more compactly. The main problem is that one must explicitly specify which operations and element versions refer to the same object, which happens very frequently in our application. Additionally, the order of editing operations must be specified by enforcing an order between time stamps. Furthermore, MEdit4CEP displays all event properties at all times, which is not necessary for the given application, where the same two event types are used over and over. Note that we left out properties not relevant to the small pattern presented, and more properties are relevant in general. Both diagrams reach the limit of what is comfortably displayable on one screen even for this very small example. We therefore come to the conclusion that the presented unspecialized visual languages are not applicable for larger patterns within our application.

VisPaRec differentiates itself through several design choices of which we regard the following ones as most significant.

1. VisPaRec uses swim lanes to keep track of element versions as well as editing operations regarding the same object. The other two considered visual languages use the guard concept to model this. While guards prevent introducing a new concept, they require encoding the semantic of referring to the same object numerous times.
2. VisPaRec gives editing operations a specialized visualization, whereas Strelka and MEdit4CEP use their respective guard syntax. Thereby, VisPaRec can reduce the number of elements to a more manageable amount and differentiates the semantically very different editing operations visually as well.
3. VisPaRec colors sequence constraints and property constraints differently, which again keeps semantically different concepts also visually distinguishable.

Regarding graphic economy, Strelka uses a very low number of different symbols. VisPaRec does introduce more symbols than Strelka, as it is necessary to cover the range of semantic constructs deducted from the metamodel in Fig 2. MEdit4CEP uses some symbols that we find too excessive for the application, and simply inflate the number of symbols. Examples for this are the element naming, the differentiation between element and properties, as well as the numbers identifying the inputs of the = relation (even though it does not matter since = is a symmetric relation).

#### 4.3.2 Evaluation of textual event processing languages

To analyze the similarities and differences of common EPLs applicable for the definition of activity patterns, we discuss main language concepts on an example activity written in Rapide, DRL, and Esper EPL (cp. Section 2). The resulting specifications are shown in Listing 1, 2 and 3, respectively. For brevity and readability, we are omitting surrounding parts of the pattern specification like rule declaration, variable declarations, rules’ action part etc. and focus on the core pattern definitions.

**Listing 1**. Example activity written in Rapide.

1 EditingOperation(Element_Version is ? V3) where

2  ( ? V3.Type = “attribute”)

3 ? 01(Element_Version is ? V4, Operation_Type is “delete”) where

4  ( ? V4.ID = ? V3.ID)

5 ? 02(Element_Version is ? V5, Operation_Type is “add”) where

6  ( ? V5.Type = “class”)

7 ? 03(Element_Version is ? V6, Operation_Type is “modify”) where

8  ( ? V6.ID = ? V5.ID and ? V6.Name = ? V3.Name)

**Listing 2**. Example activity written in DRL.

1 EditingOperation( $ V3 : Element_Version, $ V3.Type == “attribute”)

2 $ 01 : EditingOperation( $ V4 : Element_Version,

3  Operation_Type == “delete”, $ V4.ID == $ V3.ID)

4 $ 02 : EditingOperation( $ V5 : Element_Version,

5  Operation_Type == “add”, $ V5.Type == “class”)

6 $ 03 : EditingOperation(V6 : Element_Version,

7  Operation_Type == “modify”, $ V6.ID == $ V5.ID,

8  $ V6. Name == $ V3. Name)

**Listing 3**. Example activity written in Esper EPL.

1 ( EO3 = EditingOperation (Element_Version.Type = “attribute”) ->

2 01 = EditingOperation (Operation_Type = “delete”,

3  Element_Version.ID = EO3.Element_Version.ID) ) and

4 ( 02 = EditingOperation (Operation_Type = “add”,

5  Element_Version.Type = “class”) ->

6 03 = EditingOperation(Operation_Type = “modify”,

7  03. ElementVersion.ID = 02.ElementVersion.ID) )

8 where 03.ElementVersion.Name = 01.ElementVersion.Name

*Assignments*. In the Listings 1, 2 and 3, assignment operators are colored blue, while comparison operators are colored red. A comparison shows that the languages differ in how they mark assignments, how they express relations to constants and how they express relations to variable parts. Rapide uses the keyword “is” for variable assignments as well as comparisons with constants. It uses “=” for comparisons between variables. DRL uses “:” for all assignments, but “=” for both types of comparison. In Esper EPL, assignments as well as comparisons use “=”. All three ways of specification are reasonable, as the separation of syntactically different operators is important but it also is valuable to keep the number of syntactical elements of a language manageable. Furthermore, Esper EPL is missing a syntax construct that allows assignments of attributes to variables. While the syntax for this can be seen in line 1 for Rapide and DRL, Esper EPL needs to refer to the attribute using the object every time. This can be rather clunky when using a sub-object often, or when referring to an attribute of a sub-object.

*Variables*. All variables are colored green in the examples. The type of editing operations like *O*1 and *O*2 must be declared on every use in DRL and Esper EPL, while Rapide declares all variables at the start of the file. Rapide, therefore, is very compact in specifications where object types are often reused. Variables are typically marked with the prefix “?” in Rapide and “$” in DRL, while Esper EPL usually does not use such a prefix. Although a prefix adds an extra character that may look confusing at first, it helps differentiating variables from constant expressions. The prefix “?” stresses the variable nature and thus has a somewhat plausible association with a variable. Anyway those are merely conventions.

*Sequencing*. All sequencing related language elements are colored purple in the examples. In Esper EPL, the sequencing of events is specified through the “− >” operator, which is used whenever the left hand side event happens before the right hand side event. It binds weaker than the “and” operator. The keyword “where” is used to filter the matches of a pattern after it is completed. This is necessary because the example activity has two concurrent streams of events that must be connected by the constraint of identical names. While Esper EPL’s syntax allows the user to specify the sequence of events in a very compact way, the user must explicitly specify every detail of the sequence. On the one hand, this makes the user more aware of the event flow than the syntax of Rapide or DRL, on the other hand, we found it surprisingly hard to get the sequencing right for examples within our application. The other two languages formulate sequencing constraints just like any other property constraint, while the internal matching order is determined by the order of the statements from top to bottom.

In conclusion, all three textual languages were able to express the example within a similar amount of space. We discuss their applicability in regards to modeling activity patterns within the context of our evaluation in Section 6.1.

## 5 Defining modeling activities by example

We now propose a semi-automated definition process that comprises three steps: (1) demonstrating the modeling activity and deriving an initial activity definition, (2) manually refining the activity definition, and (3) generating platform-specific event-processing rules from the activity definition. The following subsections describe these three processing steps in detail.

### 5.1 Step 1: Demonstrating the modeling activity

The first step is often the hardest. Therefore, providing an intuitive way for creating an activity definition rather than starting to construct it from scratch is highly desired. A state-of-the-art way to address this goal is the definition “by-demonstration”, which allows a user to simply execute a desired behavior within a familiar working environment and to automatically derive an initial description based on that demonstration. This demonstration step starts on an empty diagram of a supported model type, e.g., a UML class diagram, and comprises two phases: (1a) modeling the activity preconditions and (1b) performing the editing operations. The following paragraphs describe these two phases and the transformation rules that are applied to create an activity definition that conforms to the meta-model depicted in Fig 2.

#### Step 1a: Modeling the activity preconditions

The preconditions of a modeling activity comprise model elements that have to be present before the modeling activity may be performed. In order to demonstrate the preconditions, the user adds the model elements to an empty diagram and defines their important properties. The preconditions for our example activity can be seen in Fig 1. Before starting the activity, a class must exist containing an attribute to be extracted. While the user is modeling the activity preconditions, *Rule 1* to *Rule 4* are applied. This step leads to at least one element version for each modeled element, specifying all relevant property constraints. Once all activity preconditions have been modeled, the user approves them and thereby enters phase (1b): the demonstration of the editing operations.

**Rule 1**: Add new model element

**1** **Trigger**: *editing operation* *o* of type add for a *model element e* (*o* ∈ *O*, e∈E|o=add(e))

**2**  **create** an *element version* *v*_**0**_, where *v*_0_ specifies the state before *o*: *v*_0_ = *pre*(*o*)

**3**   **define** the non-existence of *e* by setting *v*_0_ to type *Non-Existent*

**4**  **create** an *element version* *v*_**1**_, where *v*_1_ specifies the resulting state of *o*: *v*_1_ = *result*(*o*)

**5**   **forall** non-empty properties *x* of *v*_1_, except *ID* and *Name* **do**

**6**    **define** a *property constraint* of the type (*identifier*(*x*_*i*_) = *value*(*x*_*i*_))

**Rule 2**: Modify existing model element

**1** **Trigger**: *editing operation* *o* of type modify for a *model element e* (*o* ∈ *O*, e∈E|o=modify(e))

**2**  **retrieve** an *element version* *v*_*n*_, where *v*_*n*_ is the last version of *e* preliminary to *o* (*v*_*n*_ = *last*(*e*))

**3**  **create** an *element version* ***v*_*n*+1_**, where *v*_*n*+1_ specifies the state of *e* subsequent to *o* (*v*_*n*+1_ = *result*(*o*))

**4**   **forall** properties *x* of *v*_*n*+1_ **do**

**5**    **if** value(*x*(*v*_*n*_)) ≠ value(*x*(*v*_*n*+1_)) **then**

**6**     **define** a *property constraint* of the type (*identifier*(*x*) = *value*(*x*))

**Rule 3**: Delete existing model element

**1** **Trigger**: *editing operation* ***o*** of type delete for a *model element* ***e*** (*o* ∈ *O*, e∈E|o=delete(e))

**2**  **retrieve** an *element version* ***v*_*n*_**, where *v*_*n*_ is the last version of *e* preliminary to the model element deletion operation *o*: *v*_*n*_ = *last*(*e*)

**3**  **create** an *element version* ***v*_*n*+1_**, where *v*_*n*+1_ specifies the state of *e* subsequent to the model element deletion operation *o*: *v*_*n*+1_ = *result*(*o*)

**4**   **define** the non-existence of *e* by setting *v*_*n*+1_ to type *Non-Existent*

#### Step 1b: Performing the editing operations

Upon approving the preconditions, *Rule 5* is applied. This removes obsolete element versions and editing operations and leads to exactly one element version of type *Existent* for each preexisting model element. The user then performs the editing operations of the modeling activity involving preexisting and newly created model elements. Again, *Rule 1* to *4* are applied in order to create the corresponding objects in the activity definition. To demonstrate a delete operation, a preexisting model element is deleted from the diagram. For demonstrating an add operation, a new model element can be added from the toolbox without the need to change any property. The user only needs to edit those element properties that shall be transformed into property constraints of the activity definition (*Rule 2*). Our activity recognition approach handles the undo of editing operations within the modeling tool, i.e., a user may use this functionality upon mistakes in performing editing operations and modeling preconditions without impact on the resulting activity definition. Fig 1 shows that *Class2* has been modified to express a property constraint: the name of *Class2* must be set to the name of the extracted attribute. Most of these property constraints can be derived automatically during the demonstration step by applying *Rule 6*. *Rule 6* replaces identical constant property values with references to the corresponding model elements, making the static property constraints dynamic. Fig 12 illustrates such an initial definition that was obtained by demonstrating the example activity shown in Fig 1.

The presented set of transformation rules is independent of the model type that an activity is applied to. The set is meant as a basic rule set for creating initial activity definitions according to the specified meta-model. However, the set may be adjusted or enhanced for other types of models.

### 5.2 Limitations of rule definition by example

Rule definition by example essentially uses a bit of event data, in our case a stream of editing operations, to infer matching rules. A rule can therefore be abstracted as one way to interpret correlations contained in the given bit of data. Unfortunately, the derivation process is inherently ambiguous, no matter how many demonstrations are given. As an illustrating example, two different element versions *a* and *b* may have the name “Factory”. The property constraints shown in Listing 4, as well as infinitely many other ones, would fit that state of data.

**Rule 4**: Create version for preexisting model elements

**1** **Trigger**: *model element e* that have not assigned any element version (e∈E|last(e)=∅)

**2**  **create** an *element version* ***v*_0_**, where *v*_0_ specifies the initial state of *e*

**3**   **define** the existence of *e* by setting *v*_0_ to type *Existent*

**Rule 5**: Remove obsolete versions and operations of model elements

**1** **Trigger**: *model element e*

**2**  **if** the model element *e* has been deleted already (∃v∈V:∧v=last(element(v))∧element(v)∉E)) **then**

**3**   **delete** all editing operations: *operation*(*e*) and all element versions: *version*(*e*) of the deleted model element *e*

**4**  **else**

**5**   **delete** all editing operations: *operation*(*e*) and all element versions: *version*(*e*) of the model element *e* except the last version *v*_*n*_: *v*_*n*_ = *last*(*e*)

**6**   **define** the existence of *e* by setting *v*_*n*_ to type *Existent*

**Rule 6**: Create property constraint reference by identifier

**1** **Trigger**: *element version v*

**2**  **forall** property constraints *p* of *v* **do**

**3**   **if** *value*(*p*) contains the identifier of a model element *e*, where *element*(*v*) ≠ *e* **then**

**4**    **create** a property constraint reference *p*_*r*_ pointing from *v* to the first existing version of *e*: *exist*(*e*)

**5**     **define** a logical expression of the type (*identifier*(*p*_*r*_) = *ID*) where *ID* references the identifier of *e*

**6**    **delete** *p*

**Listing 4**. Possible Constraints based on the same demonstration

1 a.name == b.name

2 a.name == “Factory” && b.name == “Factory”

3 a.name.startsWith(“Fac”) && b.name.endsWith(“tory”)

4 a.name.startsWith(“Fac”) && b.name == “Factory”

An example based rule definition must therefore aim to find rules that are simple and likely. As there can always be cases where the simple and likely interpretation is not the correct one, manual refinement of rules can be necessary. For example, the rule definer might have wanted to demonstrate rule number 3 of Listing 4, but since rule number 1 is simpler it will generally be preferred. Furthermore, a single example defines a very specific chronological order between all editing operations. As a large number of highly different demonstrations would be necessary to determine relevant sequence constraints, those must be excluded from our automatic detection. For the activity “Extracting attribute as class”, all of the relevant ID equivalencies and name equivalencies will be detected. But there is no way to know from a single example if any of the detected equivalencies are false positives. An interesting extension would be keeping some of the demonstrations to check whether a refinement done in the next step would lead to a contradiction.

### 5.3 Step 2: Manually refining the activity definition

In the second step of the process, the user manually refines the initial activity definition generated in *Step 1*. This step is required to generalize the activity definition and to add information that cannot be derived automatically. Refinements that need to be applied are of three generic types discussed below.

#### Edit constraints

Refinements can be done in order to (1) add additional property constraints that have not been captured during demonstration, to (2) relax or extend constraints that have been captured already, and to (3) add sequence constraints that restrict the order of editing operations for performing an activity if necessary. For example, during the demonstration of the activity in Fig 1, an editing operation referred to the modification of the associations’ subtype into aggregation, but the intention of the user is to also allow the subtype composition for this activity. To do this refinement, the user needs to extend the existing constraint *SubType* = *aggregation* into *SubType* = *aggregation*, *composition* (see Fig 12, *R*_*I*_).

#### Define completion defaults

Completion actions are automatically retrieved from specified editing operations and the property constraints of their resulting element versions (see Section 4). Additionally, the user can define completion defaults that ensure desired values of element properties after the auto-completion of a modeling activity. Referring to the previous example, when allowing two options for the association subtype, a completion default could define which subtype is used when auto-completing the activity (Fig 12, *R*_*II*_).

#### Merge captured editing operations

In the demonstration phase, an editing operation is captured for each performed and accepted modification of a model element. If a user edits the properties of an element in a chain of subsequent edits, multiple element versions are created for a model element. For recognizing and auto-completing the modeling activity, often not all captured editing operations and element versions are required. Multiple sequentially performed modify operations on the same model element can be merged into a single editing operation and resulting element version, defining all required constraints in one place. For example, if the user demonstrates the modification of the association by two editing operations, modifying the subtype and modifying the navigability, these can be merged into a single operation as shown in Fig 12.

### 5.4 Step 3: Generating event-processing rules

In the third processing step, a refined activity definition is transformed into event-processing rules that can be evaluated by a rule engine. This is necessary because our visual language cannot be understood by a CEP engine like Drools or Esper directly. The transformation is done in two stages. The first stage can be regarded as a preprocessing where implicit information is defined explicitly which is required for correctly working event patterns. This preprocessing is done automatically as follows.

#### Type constraints

In the visual language, information about element types is captured in the label of a swim lane. In the textual representation, these type restrictions have to be specified explicitly to ensure a correct recognition of model elements. *Rule 7* creates a type constraint for the first existing version of each model element.

**Rule 7**: Create model element type constraints

**1** **Trigger**: *model element* *e*

**2**  **retrieve** an *element version* ***v***, where *v* is the first existing version of *e* (*v* = *exist*(*e*))

**3**  **define** *a property constraint* of the type (*Type* = *type*(*e*)) for *v*

#### Connecting element versions

Element versions that refer to the same model element are grouped into a swim lane in the visual representation. This grouping requires extra effort in the textual notation. Therefore, *Rule 8* adds ID-equality constraints to all versions of a model element.

**Rule 8**: Bind element versions of a model element

**1** **Trigger**: *element versions* ***v*** of a *model element* ***e*** (v∈V,e∈E|v=version(e))

**2**  **retrieve** an *element version* ***v*_0_**, where *v*_0_ is the first existing version of *e* (*v*_0_ = *exist*(*e*))

**3**  **forall** *previous* element versions *w* ≠ *v*_0_ of *v* **do**

**4**   **cretae** a property constraint reference pointing from *w* to *v*_0_

**5**    **define** a logical expression of the type (*ID* = *ID*) expressing the equality of the model elements: *element*(*v*_0_) = *element*(*w*)

#### Preventing multi-matching

An activity may contain multiple add operations for elements of the same type, e.g., creating new classes. It is essential to ensure that the first editing operation cannot match multiple times. *Rule 9* adds an ID-inequality constraint requesting that the identifiers of such model elements are different.

**Rule 9**: Differentiate model elements of the same type

**1** **Trigger**: *element version* ***v*** resulting from *editing operation* ***o*** on an *model element* ***e*** of the type add: (v∈V,o∈O,e∈E|o=add(e)∧v=version(e))

**2**  **if** the value of the property constraint *Type* of *v* is equal to the value of an element version *v*_*j*_: *value*(*type*(*v*)) = *value*(*type*(*v*_*j*_)), where *element*(*v*) ≠ *element*(*v*_*j*_) **then**

**3**   **create** a property constraint reference pointing from *v* to *v*_*j*_

**4**    **define** a logical term of the type (*ID* ≠ *ID*) expressing the inequality of the model elements: *element*(*v*) ≠ *element*(*v*_*j*_)

**Rule 10**: Create sequence constraints to add-operations

**1** **Trigger**: *editing operation* ***o***

**2**  **retrieve** all *element versions* ***v*** of V, where type of *v* is *Existent* (*type*(*v*) = *Existent*)

**3**  **if** the operation type of *o* is add *type*(*o*_*i*_)) = *add* **then**

**4**   **forall** element versions *v*_*j*_ of *v* **do**

**5**    **create** a property constraint reference pointing from *o* to *v*_*j*_

**6**     **define** a logical expression of the type (*TimeStamp* > *Created*) expressing that *o* must be performed after *v*_*j*_ was created

#### Sequence constraints

Sequence constraints are required to ensure that preexisting model elements were not created after editing operations of the activity already took place. *Rule 10* adds a sequence constraint between all add-operations and the preexisting elements of a modeling activity.

In the second stage, a model-to-text transformation uses the preprocessed activity definition as input for generating event-processing rules. This transformation is platform-specific and will not be discussed here. A variety of event-processing rule engines may be utilized for recognizing modeling activities.

## 6 Evaluation

We decided to perform a comparative user study of VisPaRec. As a baseline for comparison, we selected the textual language Rapide. As the benefit of by-demonstration approaches in general has been already studied by other authors [25], we decided to restrict our study to the evaluation of VisPaRec in comparison to Rapide.

In this user experiment, we aim to answer the following research questions:

RQ1 How well can a user comprehend VisPaRec’s visual activity definitions in contrast to Rapide’s textual definitions? (Comprehensibility)
RQ2 Does the use of VisPaRec result in a faster and more correct definition of modeling activities compared to Rapide’s textual definitions? (Applicability)
RQ3 How do users judge the usability of VisPaRec in contrast to textual Rapide definitions? (Usability)

### 6.1 Choice of a baseline event processing language

All above discussed event processing languages are suitable for defining modeling activities (cp. Section 2 and 4.3). Both Strelka, as well as MEdit4CEP, display a large number of model elements, even for the reduced example. This is problematic because we intend to use practically relevant refactorings for the study, which are much more extensive. Based on that we doubt that the activity definition in any of the two languages scales well in space and visual complexity. We settled for a textual language as baseline for the following reasons: First, textual languages are the more standard way to work with CEP, given that many tools like Drools and Esper do not support visual languages. Second, tasks of a moderate complexity level still fit into a manageable amount of space being roughly a standard letter size.

Considering the three presented textual languages, we found that pattern definitions are rather similar syntactically (cp. Listing 1,2, and 3). We found the most noticeable syntactical difference within the event sequencing of Esper EPL. For our presented auto-completion application, the very explicit sequencing quickly resulted in complicated nested sequencing constructs. Based on that, we argue that Esper EPL will be more difficult to deal with than the sequencing syntax of Rapide and DRL.

Besides this, their largest syntactical differences lying within language parts that are not relevant to our modeling approach. Especially DRL and Rapide were very close for the given example. As Rapide is somewhat closer to natural language than the other discussed EPLs, we considered it the easier language to explain to a user who is not a CEP expert. It is also not bound to a certain rule engine or project. We do have substantial experience in writing readable Rapide code, as we used it to formulate patterns for our auto-completion prototype, which was implemented before we designed VisPaRec. We therefore decided to compare VisPaRec with Rapide.

### 6.2 Experimental set-up

#### Subjects

The participants comprised 16 researchers, PhD students, and masters students experienced in software engineering and knowledgeable in model-based development with UML. All of them were studying or working at the TU Ilmenau and had a computer science background. We recruited them through an email to university-wide mailing lists. Participants had 4 years of software and system development experience on average. Their self-proclaimed skill level ranged from low to very high, with an average of 2.4 on a scale from 0 to 4. The participants had much less experience with complex event processing, averaging at 0.75 years. No participant answered to have a very high skill level, and only a single participant had a high skill level in complex event processing. The study took place from April 21 to May 5, 2015. We recruited researchers and PhD students through an open call at the faculty of computer science. We also invited students attending our software engineering classes. In this way, we approached about 100 researchers and about 50 students. We required the participants to have experience in software development or complex event processing. There were no dropouts during the study. Verbal informed consent was obtained from all participants. Ethics approval was waived by the Vice President for Research of the Technical University of Ilmenau as the responsible department.

#### Procedure

The experiment was controlled using a computer that presented instructions and recorded times for completing tasks. The computer was configured into a kiosk mode preventing participants from leaving the experimental software. Participants’ answers were gathered on a paper-based questionnaire and in a structured debriefing interview where the interviewer recorded participants’ answers. First, participants filled-in an initial part on the questionnaire that inquired about a subject’s experience and skill level in system and software development, model-based development, UML modeling and complex event processing. Second, each participant was asked to study the extensive tutorial on defining modeling activities textually and visually in Rapide and VisPaRec. Third, participants were given two different activity specifications, one in Rapide and one in VisPaRec, and had to answer six questions that required understanding these specifications. Fourth, each participant had to specify an activity pattern from scratch and had to refine another one. Fifth, in a structured interview we asked subjects to rate the usability of VisPaRec’s seven definition concepts (cp. Section 4.1) in contrast to their representation in Rapide. Sixth, subjects were structurally interviewed by the experimenter about their impressions and comments regarding the visual definition language and the experiment in general. Each interview sequentially inquired about the seven concepts C1–C7 (cp. Section 4.1) and how difficult a participant perceived their comprehension and application in both notations. We used the interview guide contained in the supplementary material. Seventh, we asked participants for general feedback and improvement ideas.

#### Tasks

The experiment comprised VisPaRec and Rapide definitions of four common modeling activities: “Extracting attribute into class” (also seen in Fig 12), “Resolving many-to-many association”, “Replacing association by interface”, and “Resolving association class”. These patterns describe typical model refactorings, using five up to eight different model elements to be edited zero to two times. Therefore, they generate multiple element versions related to the same model element. Participants were given seven questions and four corresponding multiple-choice statements per question and asked to check the correct statements. The questions R1–R6 dealt with deriving the number of model elements, identifying the number of model elements existing at the start and end of the pattern, identifying and classifying the editing operations, detailing whether specific elements had certain completion actions, recognizing connections between model elements as well as understanding property constraints. These tasks aimed to establish an appropriate background knowledge of the textual as well as the visual representation before applying these notations in two tasks W1 and W2. While W1 required to construct an activity definition, W2 asked for the modification of an existing definition.

Participant’s tasks and VisPaRec’s main design decisions (see Section 4.1) are related as follows:

R1 is directly influenced by the swim lane concept, so is R2.
R3 asks for the type of editing operations and therefore interacts with the decision to encode the types visually.
R4 interacts with properties, sequencing, swim lanes and editing operation types.
R5 asks participants about property constraints, editing operations (default values) and swim lanes.
R6 interacts with all of the design choices, as it requires the participants to understand editing types, properties, sequencing and their life cycle.

In the same manner, the writing tasks W1 and W2 require interaction with all of the presented decisions.

In all tasks, the duration was dominated by the effort to understand and solve the problem, not by the physical actions made. This is due to the tasks being difficult but not requiring a lot of actions to complete: R1–R4 each required the ticking of a box, R5 and R6 required to tick two boxes. W1 is the task with the most physical effort by far, requiring the participants to finish a started activity definition. One group was required to write up to 600 characters while the other group drew a small diagram of less than 20 elements as well as less than 15 connections between them. The task W2 only required correcting a given activity definition by striking and correcting the parts participants saw as incorrect. Therefore, the amount of writing work does not have a substantial influence on the duration of tasks.

#### Treatments and measures

The experiment had one independent variable, the definition language, and two treatments, the visual and the textual representation of an activity definition in VisPaRec and Rapide, respectively. Subjects were randomly assigned to one of two groups. The comprehension tasks (R1–R6) always were related to a visual or a textual pattern definition. One group answered questions R1, R3 and R5 for the visual definition in VisPaRec and the other questions regarding the definition in Rapide, while the second group answered R1, R3 and R5 for the definition in Rapide and the remaining for the visual definition in VisPaRec. For the application tasks, the first group performed W1 using Rapide and W2 using VisPaRec, while the other group did W2 using VisPaRec and W1 using Rapide. The comprehensibility and applicability were measured quantitatively in terms of time to solve a task and correctness of the result. We assessed usability qualitatively within structured interviews, where participants were asked for a comparative rating of activity definition concepts on a Likert scale from 1 (very hard) to 4 (very easy).

### 6.3 Results and discussion

#### Comprehensibility (RQ1)

Table 2 shows the results that participants achieved in the six comprehension tasks for the textual as well as the visual language. The results of a two-tailed t-test with independent samples and unequal variances are shown on the right. The upper part of the table shows the average time needed to perform the tasks, while the lower part shows the achieved correctness. The average correctness for participants working with VisPaRec was 72.0% and 60.8% for those working with Rapide. The time needed varied greatly between questions and methods. On average, comprehending VisPaRec activity patterns was 22.5% faster than comprehending their Rapide equivalent. However, there are two questions that were answered wrong more often by participants using VisPaRec than by those using Rapide (R1 and R3). These were also the questions where participants had the highest time saving using VisPaRec. We hypothesize that there is in fact a time-correctness trade off. R1 and R3 simply ask the participants to count model elements/editing operations, therefore they might have seemed misleadingly easy and participants may have jumped to a conclusion too fast. The textual description of Rapide may have forced participants to examine the activity patterns more closely, giving them more time to think about the question as a side effect. Overall, participants working with VisPaRec worked faster and more accurate on average. We suppose that the main reason for improvement is the lifeline concept, because it displays clearly which element versions belong to the same model element. This information is rather hard to grasp without lifelines, because the user must follow a chain of ID-equivalencies to group the different element versions together.

**Table 2:** Average time spent and correctness achieved in performing the comprehension tasks R1–R6.

Task	VisPaRec mean (±sd)	Rapide mean (±sd)	Difference Visual	Statistical test	
**Time [s]**					
R1	99.9 (± 40.3)	338.3 (±170.9)	-70.5%	0.007	**
R2	151.5 (± 95.9)	218.4 (±102.7)	-30.6%	>0.05	n.s.
R3	92.6 (± 85.3)	213.5 (± 92.7)	-56.6%	0.023	*
R4	402.6 (±289.0)	247.1 (±142.9)	62.9%	>0.05	n.s.
R5	142.8 (±57.8)	311.2 (±154.4)	-54.1%	0.024	*
R6	319.0 (±176.5)	229.8 (± 72.3)	38.8%	>0.05	n.s.
∑	1,208.4	1,558.3	-22.5%		
**Correctness [%]**					
R1	50.0 (±0.50)	62.5 (±0.48)	−12.5%	>0.05	n.s.
R2	87.5 (±0.33)	50.0 (±0.50)	37.5%	0.124	n.s.
R3	75.0 (±0.43)	87.5 (±0.33)	−12.5%	>0.05	n.s.
R4	50.0 (±0.50)	37.5 (±0.48)	12.5%	>0.05	n.s.
R5	93.8 (±0.17)	81.3 (±0.24)	12.5%	>0.05	n.s.
R6	75.0 (±0.35)	45.8 (±0.18)	29.2%	0.08	*
Avg.	72.0	60.8	11.1%		

#### Applicability (RQ2)

Table 3 shows mean, standard deviation, difference in percent, effect significance and the results of a two-tailed t-test with independent samples and unequal variances for both application tasks and notations. The upper part of the table shows the average time that participants spent on solving the task, while the lower part shows the achieved correctness. Constructing activity definitions fully manually (task W1) is not the intended use case of our visual definition language, as the demonstration step can create an initial definition (see Section 5.1). However, W1 was intended to allow for a direct comparison with the writing of definitions in Rapide. Defining the described modeling activity in VisPaRec reduced the required time significantly, on average by 43% (cp. Table 3). Task W2 required participants to understand a given activity definition and to modify certain editing operations and related information. This understanding and adjusting is a common use case especially when creating definitions by example. In this task, subjects working with VisPaRec were on average 47% faster than subjects working with Rapide. These results show that the use of VisPaRec can reduce the time for defining and editing modeling activities significantly. Concerning the correctness, no significant effect could be determined. Table 3 shows that subjects achieved on average almost equal correctness results with both methods. A possible reason could be that all subjects where new to the definition of event patterns for recognizing modeling activities. The measured incorrectness may rather reflect a missing familiarity with the topic than a difference in the two representations. This part of the research question can only be answered properly in a more extensive long-term study.

**Table 3:** Average time spent and correctness achieved in performing the application tasks W1 and W2.

Task	VisPaRec Mean (±sd)	Rapide Mean (±sd)	Difference Visual	Statistical test	
**Time [s]**					
W1	716.6 (±114.7)	1,170.9 (±284.7)	-43%	0.0034	**
W2	772.7 (±256.7)	1,483.6 (±336.4)	-47%	0.0015	**
**Correctness [%]**					
W1	78 (±0.15)	77 (±0.20)	1%	>0.05	n.s.
W2	72 (±0.25)	72 (±0.19)	0%	>0.05	n.s.

#### Usability (RQ3)

Table 4 aggregates users’ qualitative ratings of VisPaRec’s seven core concepts in contrast to their representation in Rapide in terms of usability (cp. Section 4.1). The results of a two-tailed t-test with independent samples and unequal variances are shown on the right. Participants rated each concept on a Likert scale from 1 (very hard) to 4 (very easy). The visual representation of model elements (C1) significantly effects the comprehensibility (cp. Table 4). Finding out what elements are comprised in a modeling activity using VisPaRec’s representation was rated as very easy. In contrast, subjects reported that performing this task in Rapide is very hard because information about model elements must be retrieved by understanding textual constraint statements. A similar effect is visible for element versions (C2). This is understandable because comprehending the Rapide representation requires resolving references in mind to find out which versions elements undergo throughout an activity. Especially the visual grouping of evolving element versions into one element swim lane was reported as highly beneficial by most subjects. The comprehension of (C3) was reported as very easy in VisPaRec, but also as easy in Rapide. This is much less of a difference than regarding C1 und C2. We hypothesize that the reason for this finding may be that a well structured textual definition makes it easy to find the operation types “add”,“modify” and “delete”.

**Table 4:** Usability of activity definition concepts (cp. Section 4).

Concept		VisPaRec mean (±sd)	Rapide mean (±sd)	Statistical test	
C1	Model Element	**very easy**	**very hard**	< 0.001	***
		3.88 (±0.34)	1.56 (±0.73)		
C2	Element Version	**very easy**	**very hard**	< 0.001	***
		3.69 (±0.48)	1.50 (±0.73)		
C3	Editing Operation	**very easy**	**easy**	< 0.001	***
		3.88 (±0.34)	2.81 (±0.83)		
C4	Property Constraint	**easy**	**easy**	> 0.05	n.s.
		2.97 (±0.78)	2.62 (±0.96)		
C5	Sequence Constraint	**easy**	**hard**	< 0.001	***
		3.12 (±0.62)	1.81 (±1.05)		
C6	Completion Action	**easy**	**hard**	0.013	*
		3.25 (±1.00)	2.31 (±1.01)		
C7	Completion Default	**very easy**	**hard**	< 0.001	***
		3.75 (±0.45)	1.94 (±1.06)		

Nevertheless, subjects liked the clearly distinguishable visual representation of editing operations. Also not significant was the effect of VisPaRec’s visual representation of property constraints (C4). One reason is that static property constraints are represented textually in both Rapide and VisPaRec, and thus the representations are rather similar. Another reason is the representation of dynamic constraints as connectors. Subjects liked to see what model elements are related instead of resolving textual references but struggled initially with tracing the connectors from source to target. This was reported as a question of familiarization. In addition, suggestions were made to support the usage of connectors in active editors through highlighting and out-fading on “mouse-over”. This was also mentioned for sequence constraints (C5). In contrast, subjects rated the Rapide representation of sequence constraints as hard to understand. On average, completion actions (C6) were rated to be easily comprehensible in VisPaRec, but hard to understand in Rapide. It can be seen in Table 4 that both ratings have a high standard deviation, meaning that subject had very different views on it. While some subjects liked to be able to automatically retrieve completion actions, reducing redundant information, the other part liked the clear overview of writing down completion actions explicitly. We believe that eliminating redundant information in an activity definition can reduce error-proneness in the long run. Completion defaults (C7) were understood very easily in VisPaRec. The visualization of completion defaults separated from property constraints was reported as very beneficial. In contrast, defining them textually within completion actions makes it hard to distinguish between conditional and default values. When we asked participants whether they prefer reading textual languages or visual languages in general, 94% of the participants preferred reading visual activity definitions, 84% of participants preferred writing visual activity definitions over textual ones. Participants also gave us valuable feedback for future iterations of the language. Most feedback referred to visual details, e.g., one participant found the orange color of time constraints rather close to the red we use for deletion operations, while another participant advised to make the arrow starting symbols more clear in favor of color blind users. Participants also envisioned VisPaRec to be supported by active editor features, like sorting swim lanes by type and collapsing swim lanes as well as element version stacks.

### 6.4 Limitations

The experiment aimed to evaluate VisPaRec in comparison to the generic textual language Rapide for defining complex event-processing rules. We chose Rapide as a platform-independent textual definition language for the reasons elaborated in Section 6.1. While the fundamental concepts of CEP are represented across all event-processing languages, we cannot claim that our results are generalizable across all those languages, even though we do not expect substantially different results regarding different general purpose EPLs. Still, this can only be confirmed by further experiments to study other languages. Additionally, our visual language should be also compared to a textual domain specific language which is specialized for the definition of activity patterns. As we are not aware of any language specifically built for this domain, such a comparison is impossible at this time. We consider engineers, working in a model-driven software and systems development process as main audience for the proposed approach. Therefore, we requested that subjects participating in our experiment had to fit this role. All subjects had a mature level of education and at least some experience in model-driven development and UML modeling. The tasks used in the experiment were based on modeling activities commonly known from literature and often occurring in practice. However, the low number of data points does not allow to draw general conclusions. To ensure that subjects had a consistent fundamental knowledge in concepts of the experiment, we asked all subjects to work through an extensive tutorial that also included different comprehension tasks. We expect that replications of the experiment under the described conditions will offer similar results, leading to similar findings. For that purpose, we provide our material in a replication package (accessible at https://dataverse.harvard.edu/dataset.xhtml?persistentId=doi%3A10.7910%2FDVN%2FFTPF3Z).

## 7 Conclusions and future work

This paper proposed a novel way to define event-processing rules for recognizing and auto-completing modeling activities during model-driven software and systems development. By using VisPaRec, event-patterns and corresponding completion actions can be graphically modeled instead of written as textual patterns. Along with this visual language, we proposed a three-step semi-automated activity definition process. This process builds upon a by-example step that provides the user with an initial activity definition to be refined. After manual refinement, required event-processing rules can then be automatically generated. A user experiment showed that participants achieved on average higher correctness in less time when asked to understand visual activity definitions and were able to define and edit activities in significantly less time (-43%, -47%) at comparable correctness. When asked about the usability of the approach, participants rated the visual activity definitions as easy to very easy to understand in contrast to the textual baseline definition language Rapide. Almost every participant (94%) preferred reading visual activity definitions over textual. Although we embed the language into a by-example approach, 81% of the participants would also prefer writing visual definitions in a solely manual manner. The approach has been implemented as an add-in to the Sparx Enterprise Architect modeling tool [34]. Screenshots of the definition process are part of the supplementary material. Interviews with study participants provided various ideas for additional supporting functionality in this process.

Future work will comprise the evaluation of the prototype in a larger industrial study to gain more results, especially concerning the capability of reducing error-proneness. Another interesting extension point would be an evaluation of the applicability of VisPaRec’s main design choices to general-purpose CEP languages. The concept of special time and ordering constraints may be applicable as-is. However, the concept of swim lanes most likely needs to be generalized in order to support other attributes than object identification and to be useful in different scenarios. Regarding the activity definition by example, presumably higher accuracy can be achieved through the use of multiple demonstrations. We currently evaluate graph-based methods in order to support an arbitrarily amount of definitions and make the method more flexible. Another possibility is to implement VisPaRec as an interactive editor that could supply dynamic features, such as those desired by some participants of our study.
